# Numerical Investigation of Mechanical Performance and Micro-Structure Failure of Polymer-Fiber Reinforced Sand

**DOI:** 10.3390/polym15234528

**Published:** 2023-11-25

**Authors:** Runqi Zhang, Guojiao Huang, Zezhuo Song, Jiaqiang Zheng, Peng Wu, Chenyang Zhang, Yipin Lu, Zhengjie Wang, Chengjiang Dai

**Affiliations:** 1 School of Earth Sciences and Engineering, Hohai University, Nanjing 210098, China; 2 Geoscience Paris-Saclay, Paris-Saclay University, 91405 Orsay, France

**Keywords:** soil reinforcement, strength characteristics, reinforcement mechanism, numerical test

## Abstract

Natural sand has a loose and porous structure with low strength, and is prone to many geoengineering problems that cause huge losses. In this study, an organic polymer-polymer-fiber blend was used to improve the strength of sand. Using a series of laboratory and numerical simulation tests, researchers have investigated the microdamage behavior of an organic polymer and fiber-treated sand in various types of mechanical tests and explored the improvement mechanism. The results showed that the polymer- and fiber-treated sand enhanced the integrity and exhibited differential damage responses under different test conditions. The increase in polymer content induced uniform force transfer, leading to a wider range of particle motion and crack initiation, whereas the fibers adhered and confined the surrounding particles, inducing an arching force chain and dispersive/buckling cracking. Polymer- and fiber-treated sands increased their energy-carrying capacity and improved their energy release, which affected the damage characteristics. Organic polymers, fibers, and sand particles were wrapped around each other to form an effective interlocking structure, which enhances the integrity and mechanical properties of sand. This study provides novel ideas and methods in the polymer-fiber composite treatment of sand in the microscopic field.

## 1. Introduction

Sandy soil is characterized by a loose and porous structure, and its strength is low in its natural state, which may cause many geoengineering problems and considerable losses to the national economy and people’s daily lives [1]. Therefore, sandy soil needs to be improved and reinforced [2,3,4]. Traditional methods of sand soil reinforcement mainly use waterfall sand, vibratory flushing, and rubble pile methods [5] Such hard protection measures can effectively reinforce sandy soils, but they are usually consumable, expensive and have high labor costs. Therefore, in recent years, choosing a more economical and effective reinforcement method has gradually become the focus of research by scholars at home and abroad.

Organic polymers, a new type of environmentally friendly materials, can effectively maintain soil moisture, increase soil porosity, and improve soil nutrient utilization, demonstrating great potential for soil improvement [6]. Through unconfined compressive strength and triaxial compression tests, Cabalar et al. [7] found that the mechanical strength of sand improved notably with xanthan gum.

Zhang et al. [8] used a variety of composite polymers to investigate their enhancement effects on soil water retention, and erosion resistance h, and the results showed that the rigid structures of the polymer chains enhanced the structural stability of the soil.

To understand the potential mechanisms behind the improvement of soil using organic polymers. Many researchers have focused on this phenomenon using technologies such as scanning electron microscopy (SEM), X-ray diffractometry (XRD), and numerical simulation software. Liu et al. [9,10,11] conducted microstructure tests on polyurethane curing agent-modified sandy soil and the results showed that the polyurethane curing agent mainly improved the sandy soil through filling, wrapping, bridging, and other methods, effectively improving its permeability resistance and mechanical strength characteristics.

Zhang et al. [12] investigated the changes in water-holding properties and microscopic mechanisms of biopolymer-treated sandy soils over a wide suction range by scanning electron microscopy and presented a prediction model of the soil–water retention curve that considered the biopolymer ratio.

Che et al. [13,14] used environmentally friendly composite materials to improve sandy soils. Through a series of laboratory and numerical experiments, they studied the effects of different organic polymer contents and sand densities on the mechanical strength and failure behavior of improved soil. They observed that polymer materials could effectively improve the erosion resistance and stability of sandy soils.

Fibers are commonly used soil reinforcement materials that can remarkably improve the physical and mechanical properties of soil [15]. After fibers are added to sandy soil, they form an interlocking network structure within the soil, which notably improves the mechanical strength of sandy soil [16]. Jairaj et al. [17] used naturally grown sisal fibers to improve sandy soil and found that the addition of the fibers significantly improved the mechanical strength of the soil, with an optimal sisal fiber content of 2%. Xu et al. [18] conducted direct shear tests on polypropylene-, glass-, and basalt-fiber-reinforced sand using large-scale direct shear tests to compare and study the shear strength and shear expansion phenomenon of reinforced sand under different fiber-content conditions. Reddy et al. [19] conducted California bearing ratio (CBR) tests on black cotton soil reinforced with different amounts of sisal fibers, and the results showed that the CBR value of the reinforced soil was the highest when the amount of sisal fiber was 0.75%. Estabragh et al. [20] conducted triaxial and consolidation tests on palm fiber-reinforced clay, and the results showed that the addition of palm fibers improved the shear strength and compressibility of the sample.

Researchers have begun to explore the mechanism and model establishment of fiber-reinforced soils.

Zenon et al. [21] analyzed the stress-strain characteristics of fiber-reinforced sandy soils by means of triaxial drainage compression tests and found that tensile strains had to be generated in order for the fiber reinforcement to be demonstrated.

Sen et al. [22] used a combination of laboratory and numerical experiments to analyze the mechanical strength of rammed soil walls reinforced with natural and artificial fibers. Ganiev et al. [23] found through laboratory and numerical experiments that fibers inhibit the development of soil anisotropy and have advantages in increasing soil mechanical strength and improving geological structure stability.

Researchers have found that the improvement effect, cost, and construction method of single materials such as fibers and organic polymers on sandy soil have certain limitations. Considering the requirements, they have combined fibers and organic polymers into composite materials to improve sand [24]. Ayeldeen et al. [25] used liquid curing agents and fibers to reinforce soft soil. Rivera et al. [26] compared and analyzed the effects of improved soil physical properties by mixing polypropylene and wool fibers with natural curing agents to form two composite materials. Bai et al. [27] observed through experimental research that an increase in the content of polypropylene fibers and organic high-molecular polymers can enhance the compressive strength of sand while improving the failure mode of improved sand.

Currently, most research on the mechanism of composite material reinforcement of soil is conducted worldwide using electronic measurement instruments such as SEM and FTIR, whereas, the research on numerical simulation experiments is limited. Yuan et al. [28] used glass fibers and modified polyvinyl alcohol polymers to reinforce granite residual soil. The SEM results showed that the bonding between the granite residual soil solidified with a high polymer concentration and the soil particles was stronger. The tensile strength of the glass fibers increased with the addition of the glass fibers. Zhao et al. [29] explained the mechanism of microbial-induced calcium carbonate precipitation and fiber-combined reinforcement of sandy soil; SEM images showed that the fibers were embedded in precipitated calcium carbonate crystals filled with voids.

In this study, organic polymers and polypropylene fibers were used for the composite modification of sandy soil. Laboratory and numerical tests were conducted to analyze the changes in the improved sandy soil and the effects of the curing agent and fiber contents on the physical strength characteristics of the improved soil were discussed. The changes in the strength characteristics, failure modes, and development of microcracks during the deformation and failure of the composite improved sand under different variable combinations were systematically summarized. Based on the comprehensive experimental results, the microstructure and improvement mechanism of the composite-improved sand were determined. It provides some theoretical support for the composite-modified sand.

## 2. Materials and Methods

### 2.1. Materials

#### 2.1.1. Organic Polymer

**Figure 1:**
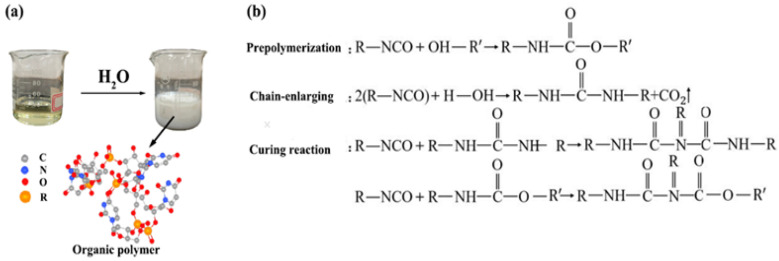
Structure (**a**) and preparation process (**b**) of organic polymers.

The organic polymer used in the experiment is a light-yellow clear liquid, which contains repeating amino formate structural units (-R-NH-CO-O-R). When it comes into contact with water, it forms a white viscous emulsion (Figure 1a). The preparation process is shown in Figure 1b: (1) Prepolymerization reaction, isocyanate reacts with polyols to form amino formate; (2) Chain-enlarging, isocyanate reacts with water to form urea and carbon dioxide, increasing the relative molecular weight; (3) Curing reaction, isocyanate further reacts with urea or amino formate to form a crosslinked structure.

#### 2.1.2. Polypropylene Fiber

**Figure 2:**
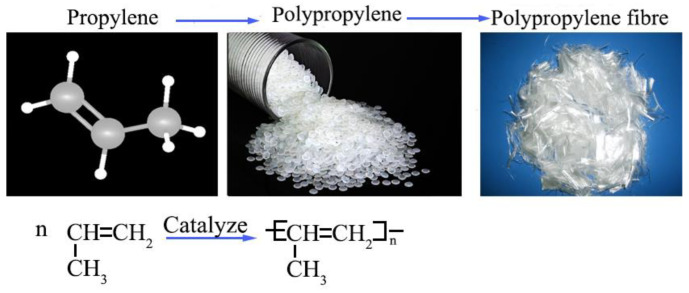
The used polypropylene fiber.

Polypropylene fiber is purchased from Shandong Tongying New Materials Co., Ltd., Tai’an City, China, which is a white bundle-shaped monofilament fiber synthesized from polypropylene and other materials through artificial synthesis (Figure 2). Polypropylene fiber is colorless, odorless, and non-toxic. It is insoluble in water and organic liquids at room temperature, but soluble in aromatic hydrocarbons. Soil improved with polypropylene fiber has good resistance to erosion, fatigue, seepage, and cracking.

#### 2.1.3. Sand

The sand used in the experiment was taken from the vicinity of Xinmeng River in the Taihu Lake Basin, Changzhou City, Jiangsu Province, China. The obtained sand samples were dried and sieved through a 2 mm sieve. According to the “Standard for Geotechnical Test Methods” (GB/T 50123-2019), various basic physical parameters of the sand were tested, and the main properties of the soil sample are shown in Table 1.

### 2.2. Laboratory Test Scheme

**Figure 3:**
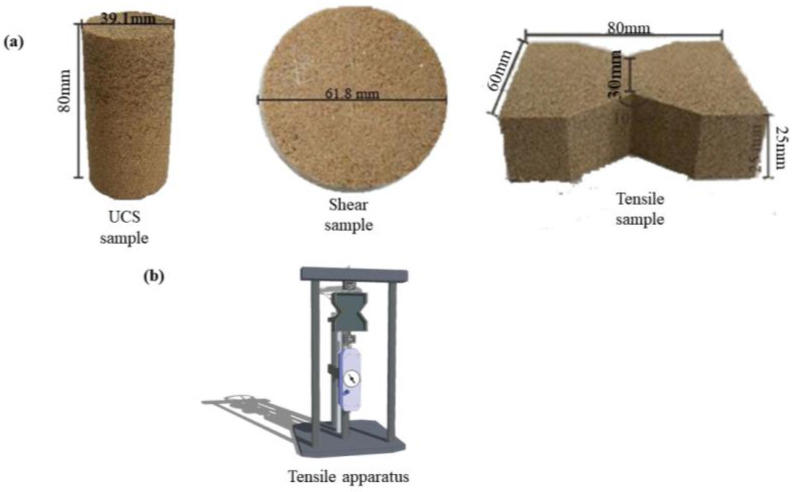
The samples for mechanical tests (**a**) and tensile apparatus (**b**).

According to the preliminary test results, the length of polypropylene fibers was 18 mm, and the fiber mass fraction was 0.2%, 0.4%, 0.6%, and 0.8%, respectively. The organic polymer mass fraction was 1%, 2%, 3%, and 4%, respectively. The experiments were conducted according to an orthogonal experimental design. According to the above experimental plan, appropriate amounts of water, sand, polypropylene fibers, and organic polymers were weighed, and uniaxial compression (UCS), shear (DS), and tensile (DT) samples were prepared using a static compaction method, as shown in Figure 3a. Standard instruments were used to conduct unconfined compression tests (ASTM D2166) [30] and direct shear tests (ASTM D3080) [31] on the improved sand samples, with loading rates and shear strain rates of 2.4 mm/min and 1.2 mm/min, respectively. In addition, a self-designed tensile apparatus was used to conduct direct tensile tests, as shown in Figure 3b.

### 2.3. Numerical Test Scheme

**Figure 4:**
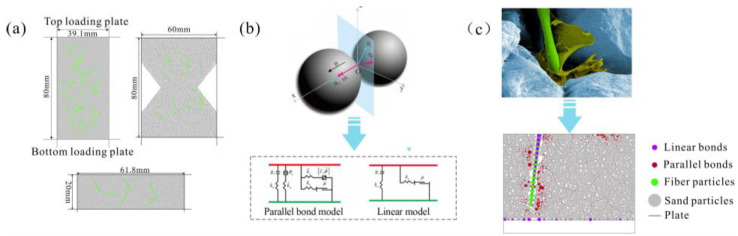
Sample numerical model (**a**), cementation model (**b**) and cementation arrangement (**c**).

The laboratory test results indicate the macroscopic mechanical strength characteristics and deformation failure characteristics of the organic polymer-fiber improved sand. However, the structural response and potential failure of the samples under load cannot be reflected by laboratory tests. Numerical tests can further analyze the potential mechanisms and evolution process of the bond failure between organic polymers and sand particles and the fiber fracture. In this study, different boundary conditions were set according to the three types of tests mentioned earlier to establish corresponding numerical models, as shown in Figure 4a. The fibers are composed of particles with a radius of 0.02 mm and are randomly distributed in the model according to different contents. (Rectangular closed walls with a size of 18 mm and a width of 0.04 mm were randomly generated in the model sample through the built-in program of PFC geometry. The center position of the bottom short side and the Angle between the long side and the horizontal plane in the four facets of the fiber wall are calculated. A series of 0.02 mm fiber particles are generated in the closed wall. According to the mechanical mechanism of fiber, the contact behavior is simulated by linear contact simulation, and the fiber particles are bonded to each other as a whole fiber. Then, the corresponding content of fibers in different positions and directions is randomly generated in the model sample repeatedly, and the particles intersecting with the wall are deleted to make the fibers evenly distributed in the model sample. Finally, the wall surface is removed and all fiber particles are detected to ensure that all fiber particles are in contact with the model sample particles).

Due to the presence of polymers, modified sand is considered a continuous medium material, and the contact between sand particles can be simulated using a parallel bonding model, as shown in Figure 4b. This model consists of two parts: the linear elastic part and the bonding part. The linear elastic part can transmit the force between particles and simulate the elastic behavior of inter-particle contact and relative displacement. The bonding part can transmit the moment and define the viscous contact behavior between particles. When either of the two parts is damaged, the bonding effect between particles is eliminated, which is similar to the behavior of actual polymer films. According to the mechanical mechanism of fibers, a linear contact model is used to simulate their contact behavior. There is no direct numerical relationship between the microscopic parameters and the macroscopic mechanical parameters of modified sand (such as modulus, peak strength, peak strain, and Poisson’s ratio), so a “trial and error” method is usually used for parameter calibration. In this process, the numerical simulation follows the same procedure as laboratory experiments, with an initial setting of microscopic parameters and continuous iteration until the macroscopic parameters and failure mode of the experimental model are consistent with the laboratory results. The detailed parameter settings are shown in Table 2.

### 2.4. Verification

In numerical experiments, the discrete element software PFC2D 5.0.28 was used to comprehensively analyze the distribution of contact force chains, crack propagation modes and distribution and energy evolution process of organic polymer-fiber composite improved sand under axial load, shear load and tensile load under different conditions.

**Figure 5:**
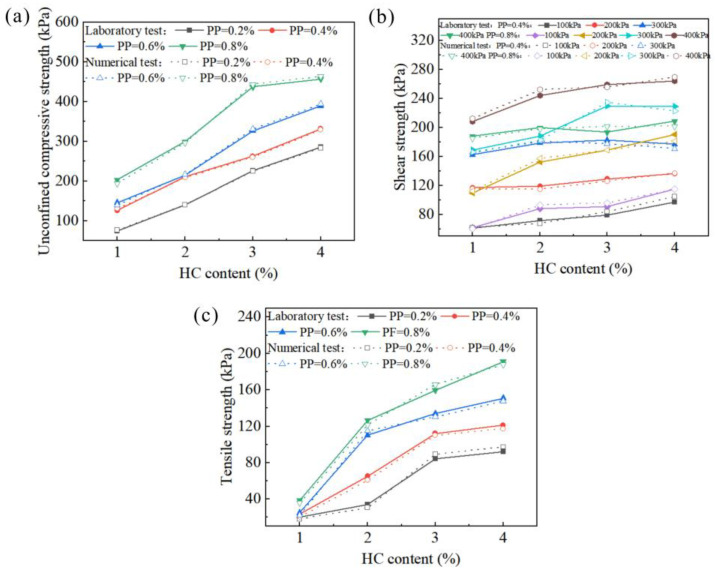
Comparison of the results of compressive (**a**), shear (**b**) and tensile (**c**) laboratory tests and numerical tests.

Compare the data results obtained from the calibration of numerical experiments with the laboratory test results, as shown in Figure 5. It can be seen that the data results have a significant degree of similarity. The macroscopic cracks in the numerical model are basically consistent with the failure morphology and distribution of the actual specimens. The reliability of the calibration results of the simulated experiments in Table 2 has been fully verified.

## 3. Results

### 3.1. Breakage of Inter-Particle Bonding

**Figure 6:**
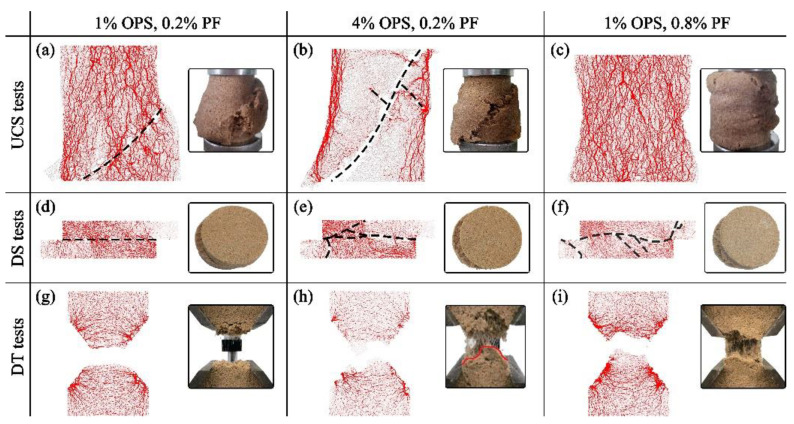
Inter-particle bonding breakage diagram of the typical specimens:. UCS tests of (**a**) 1% OPS with 0.2% PF, (**b**) 4% OPS with 0.2% PF, and (**c**) 1% OPS with 0.8% PF; DS tests of (**d**) 1% OPS with 0.2% PF, (**e**) 4% OPS with 0.2% PF, and (**f**) 1% OPS with 0.8% PF; DT tests of (**g**) 1% OPS with 0.2% PF, (**h**) 4% OPS with 0.2% PF, and (**i**) 1% OPS with 0.8% PF.

In the numerical model, randomly generated particles lead to a complex contact network and the inter-particle interactions can be represented as force chains. In other words, polymer bonding can be represented as force chains. Bond breakage occurs when the force applied between particles exceeds the force chain limit. In this study, OPS and PF differentially affected the bonding breakage of modified sand, but the effects caused by single-factor variations were similar. Therefore, the basic, high OPS content and high PF content specimens were selected as typical models to be analyzed, as shown in Figure 6. With the increasing external force, the particles rotated and moved, resulting in the main force bending chain. The micro-fractures gradually connected into large cracks, leading to concentration and depression of the force chain. Differential bond breakage patterns were observed under the three test methods with the use of organic polymer stabilizer (OPS) and polypropylene fiber (PF).

It was observed that for less OPS and PF, single concentrated (Figure 6a) or flat (Figure 6e,g) macroscopic breakages were demonstrated. In the compressive tests, high levels of polymer content generated multiple breakage paths across the specimens, because of an improved homogeneity of stress and a spread of stress concentration in diverse directions. Fewer bond breakages were identified at higher fiber content. The force chains were arranged in an arch formation to bear the load, resulting in the lateral bulging of the sample. In the shear tests, the specimen experienced breakage primarily between the blocks due to the shear box. The force chain was concentrated in the upper left and lower right corners of the specimen with the force chain between the blocks intersecting the shear surface obliquely. The increase in OPS and PF had a similar effect, intensifying the concentration and inclination of the main force chain, leading to a rough and curved breakage surface. The elevated homogeneity of the specimen results in an increased range of particle motion. In the tensile tests, the increase in additives similarly led to a significant irregularity of the breakage surface. This illustrates the strong interlacing effect of the polymer membranes and fibers and the enhancement of local stability.

**Figure 7:**
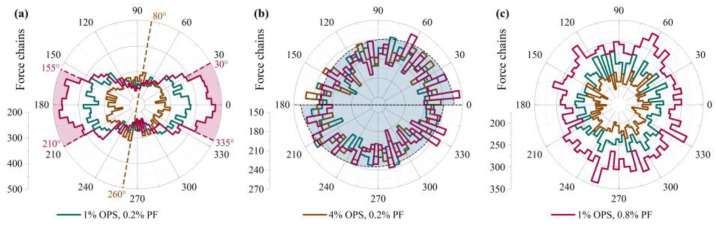
Distribution Results of Contact Forces during Failure Stages under Different Tests: (**a**) UCS tests, (**b**) DS tests, (**c**) DS tests.

The comparative results of force chain distribution in the breakage stage under different tests were illustrated in Figure 7. The compressive test had the most notable influence on the structure of force chains. With increasing polymer content, the force chains tend to evolve from a primarily horizontal distribution to a vertical one in the direction of 80°–260°. This is due to the division of multiple fracture paths leading to much breakage of the force chain in the horizontal direction. With increasing fiber content, the arch arrangement facilitated the distribution of force chains in a horizontal orientation between the directions of 30–210° and 155–335°. For the shear test, the force chain distribution is staggered along 0–180° because the shear box specifies the breakage surface. The force chain is inclined to the direction of force. For the tensile test, the specimens were stabilized after the breakage, and the force chains were predominantly distributed longitudinally. The force chain structure is similar for each additive, except for the quantity variation. The analysis indicates that more OPS results in a greater range of bonding breakage and consequent uniform centripetal shrinkage of the rose diagram. On the other hand, more PF increases the roughness of the breakage surface but decreases fracture range, resulting in uniform expansion of the rose diagram.

### 3.2. Microcracking Response

**Figure 8:**
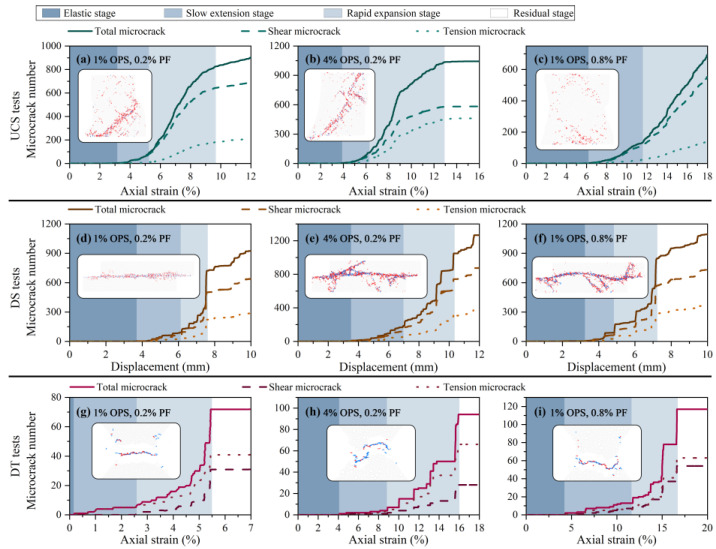
Cracking curves and distribution of each specimen.

Bonding breakage can be reacted as inter-particle microcracks, which serve as the foundation for macroscopic fracture. The microcrack propagation mode is shown in Figure 8. Crack progression consists of four stages: the elastic stage (I), the slow extension stage (II), the rapid expansion stage (III), and the residual stage (IV). When in Stage I, no microcracks are formed, signaling that the specimen is still structurally sound. With loading, including compression, shear and tension, stage II is then initiated. Microcracks developed randomly in compressive samples, whereas in shear and tensile samples, they primarily originated on the respective surfaces under stress. Further loading resulted in the aggregation and escalated number of microcracks, leading to Stage III development. Eventually, the modified sand’s internal structure was compromised through penetration, the maximum bearing capacity was attained, and microcrack progression slowed down substantially and stabilized in Stage IV.

In both UCS and DS tests, it was observed that shear microcracks occurred before tension microcracks, comprising the majority of damage to the specimens. Such microcracks primarily accounted for the harm caused. The differential displacement caused by the shear of the particle bonding creates the tensile stress field, which is the cause of tensile cracks. The opposite was observed in the case of tensile tests. The distribution and morphology of macroscopic cracks were controlled by the complementary expansion of shear and tension microcracks. Furthermore, the total number of cracks increased with increasing additive content, except for the multi-fiber compressive specimens. This can be attributed to the OPS and PF treatments which improved the homogeneity of the modified sand, causing a larger scope of structural instability. It can be corroborated by the multifocally occurring and flexure-expanding microcracks in Figure 3. Whereas the multi-fiber compressive specimens did not enter stage IV within 20% strain rate (Figure 3c). The dispersed distribution of microcracks indicates that the reinforcing effect of the fibers effectively enhanced the deformation resistance of the modified sand and reduced the microcrack generation. From Figure 3g, the basic specimen had difficulty resisting tension, with bonding breakage occurring early during loading, and fracturing at only 5.5% axial strain. In contrast, both more treated specimens exhibited good tensile resistance (Figure 3h,i). Each of them experienced cracking at about 4% axial strain and fractured at around 16%.

### 3.3. Energy Response

**Figure 9:**
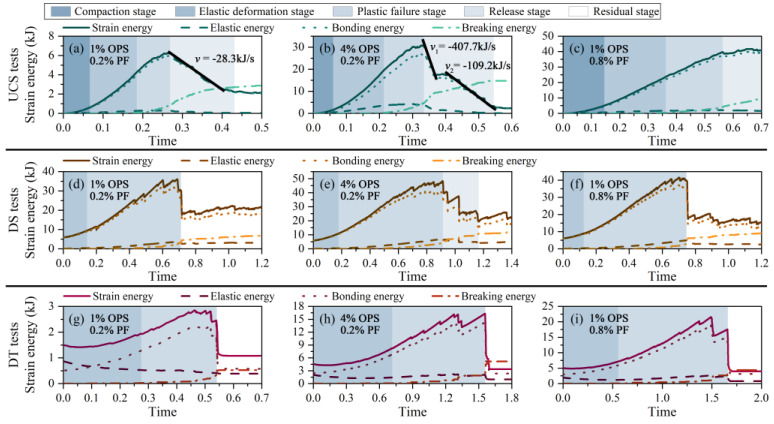
Energy evolution diagrams of each sample under different tests.

The energy evolution of each specimen was extracted as shown in Figure 9. The external input energy can be converted into the strain energy generated by the modified sand resisting deformation, which consists of the bonding energy and elastic energy of the particles. Where the bonding energy is defined as the energy of OPS forming a glue between sand particles to resist deformation by external forces and the elastic energy is defined as the energy generated by sand particles contacting and squeezing each other under external forces. The damage of modified sand under external force conditions can be regarded as the result of the breaking energy driving the particle damage. From Figure 9, there are five stages of energy transformation, which behave differently in the three tests. The evolution of each specimen based on the same test is similar, with more additives, the total energy and strain energy that the system is able to take increases.

The UCS test undergoes the most complete energy stages: compaction stage (I), elastic deformation stage (II), plastic failure stage (III), release stage (IV), and residual stage (V). In stage I, the pores gradually close, and inter-particle squeezing has an insignificant effect. Energy input accumulates mostly in the particle bonding, and the bonding energy curve aligns with the strain energy curve while elastic energy equals zero. Entering Stage II, the particles make contact and experience greater stress resulting in enhanced interlocking. The external energy is converted into elastic energy for elastic deformation. As strain increases, the bonding portion breaks down generating breaking energy and leading to Stage III, which corresponds to the slow growth of microcracks. During this process, the bonding and elastic energies increase reaching a peak at the end of the stage. Next, at stage IV, the energy kept in the system quickly releases, the strain energy decreases, and a large breakage of inter-particle bonding happens. This matches the fast growth of microcracks. Lastly, the fracture penetrates and enters Stage V. At this point, the energies stabilize, the strain energy equals the bonding energy, and breaking energy becomes the main transformed energy.

The energy release rate symbolized as v, is defined as the decreasing slope of the strain-energy curve during the release stage. The efficiency of energy release increases as the value of v decreases. The energy release rate for the modified sand with 1% OPS is −28.3 kJ/s. In contrast, the strain energy curve for 4% OPS decreases in steps and has two v values of −407.7 kJ/s and −109.2 kJ/s. This phenomenon can be explained by the observation that as the polymer content increases, the modified sand necessitates more paths to release energy. Inconsistent cracking path penetration times led to the creation of multiple low values of v. Additionally, the multi-fiber specimens exhibited higher ductility and did not suffer significant damage during axial loading, thereby avoiding entering stage V. From Figure 9b,c, more fibers resulted in a greater improvement in the efficiency of the modified sand in transforming the exogenous energy, with less enhancement of the inter-particle occlusal force (less elastic energy increment).

Owing to the constraints of the shear box, the energy induced by the volume change is poor. Consequently, during the shear test, the specimen enters directly into elastic deformation (II), plastic failure (III), and the residual stage (V) (Figure 9d). At low OPS levels, the PF cannot couple well to the particles for stabilization and the inter-particle bonds are easily broken. The strain energy initially rises steadily to a maximum, then drops sharply and proceeds directly to a stable stage V. More polymer covers this by improving the specimen integrity, allowing the input energy to be released slowly (Figure 9e Stage IV). This enhancement provided is more significant compared to that of fibers.

The energy evolution of the tension test follows a similar pattern, including elastic deformation (II), plastic failure (III), and a residual stage (V). The tensile force directly impacts the particles and inter-particle bonding resulting in high initial elastic and bonding energy. As tension progresses, particle rearrangement takes place and the elastic energy reduces till an initial stabilization is achieved. Plastic failure then occurs and breaking energy accumulation is monitored. With a complete fracture of the specimen, the curves undergo an abrupt change thus entering stage V. It was observed that both more treatments delayed the time to peak, conferring better tensile resistance to the modified sand.

## 4. Discussion

### 4.1. SEM Observations

Organic polymers comprise a large number of long polymer chains and a rich active isocyanic acid-base (-NCOB), and hydrophilic groups are easily associated with water. In the natural state, the sandy soil structure is loose with no cohesion, and the contact form of the particle is point-point. When the organic polymer emulsion fills the pores between the sand particles, the organic polymer membrane acts as a “bridge” between the sand particles, and the contact form is transformed into surface-surface adhesion. This adhesion mainly includes physical and chemical adhesion. The physical adhesion effect of the van der Waals force was enhanced by increasing the contact points of the organic polymer membrane. For sandy soil, the number of Si^−^, OH^−^, Ca^2+^, and Mg^2+^ ions are low; however, they can be combined with the active functional groups on the molecular chain of organic polymers, forming hydrogen bonds and providing chemical adhesion.

Fiber is mainly used to improve the mechanical properties of a sample through friction between the surface of the fiber and sand particles. Natural sandy soil is loosely structured and slightly cohesive, making it difficult to mix the samples directly with fibers. The organic polymer provides cohesion between the sand particles in the organic polymer sand soil. When the fibers are mixed, the interweaving permeates the sand particles and forms a three-dimensional network constraint. In addition, the fiber is provided with additional adhesion sites, beneficial to the polymer membrane packaging fiber and sand particles. The contact surface and the occlusion of the fiber and sand particles are increased, and the coupling degree of the fiber network and the sand particles is improved, which enhances the constraints of the fiber on the sand particles.

### 4.2. Different Processing of Sample Microfracture Mode

#### 4.2.1. Influence of Polymer on Microfracture Mode

**Figure 10:**
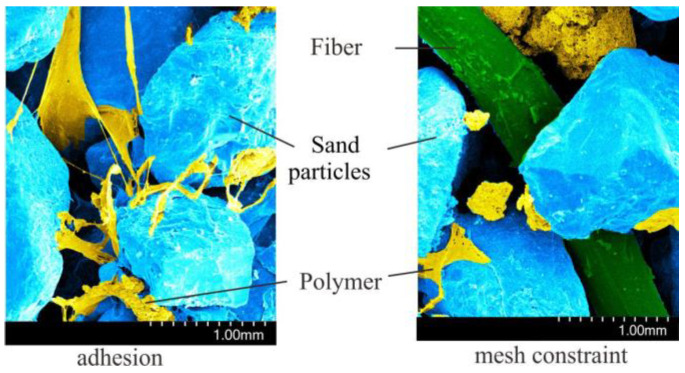
SEM images of organic polymer and fiber-modified sand.

**Figure 11:**
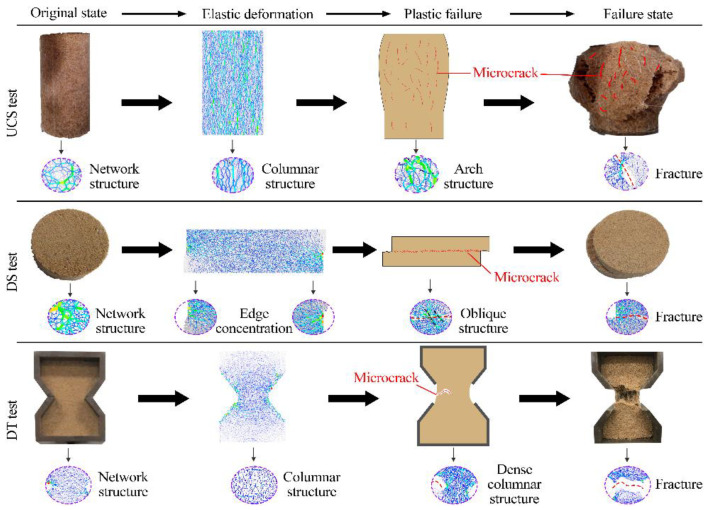
Schematic diagram of the compressive failure mode of polymer reinforced sand.

SEM images of organic polymer and fiber-modified sand are shown in Figure 10. An increase in the organic polymer content densified the contact force chain of the sample, and the line of the force chain was thick. During the loading process, the deformation process of different test samples is widely used, and the plastic failure, destruction, and residual stages are common. The damage mode mechanism is shown in Figure 11 using the compressive test as an example. As the number of organic polymers increased, the energy of each phase became non-linear, and the ratio of the peak strain to total energy gradually increased. In the elastic deformation phase, the distribution of the force chain in the sample and the force on each part of the sample were more uniform, and an increase in the organic polymer content had no effect on them. To improve the treatment of the sample case, the force of the sample case and the structure of the force chain were improved, the force chain structure evolved into the form of more branches, and the sample crack developed from a single path to a multi-path, with a lot of microcracks in the process and gradually developed from directional to non-directional. The higher the contact force chain density, the lesser bending in the coefficient of the chain due to the fact that cementation of the particle’s organic polymer membrane restricts the deformation of sand soil and the deformation of the sample is constrained. The effect of the bonding can reduce the damage to the particles and enhance the ability of the soil to resist deformation. In the failure phase of different samples, the number of shear microcracks was the major part of the total microcracks, and the shear microcracks were the main components of the sample fracture; the development of the crack was low when the increase in the organic polymer content increased the shear microcracks. The significant improvement of sand clay particles and cement damage requires more destruction paths to release energy. The number of microcracks rapidly increases, the improved sand soil is divided by multiple fissures, and the fracture surface is mixed over time, resulting in the production of several low energy release rates. The increase in organic polymer content enhances the larger external loading and deformation of the particles, and the overall microstructure stability ability is improved. The higher external load causes a greater force range, which eventually causes a greater particle movement, causing the improved sand soil to be affected by tension and increasing the number of microcracks in the final destruction.

#### 4.2.2. Effect of Fiber on Microdamage Mode

The friction and adhesion force of the fiber and soil were reinforced, and there was better coupling and cementation between the sand grains. The friction effect of the fiber and sand grain was remarkably enhanced, the displacement of the sand grain in the finite space was restricted, the clearance of the sand particles was filled, and the bonding effect of the organic polymer was enhanced. In the process of improving the sandy soil, the number of contact force chains increased, and the arch chain was arranged in a large diamond arch, which provided toughness for the improved sandy soil; the total energy increased and non- linear growth was observed. In the early stages of loading, the strain energy and consolidation increased. As the loading process continued, the rubber in the sample was partially damaged, the number of microcracks was stable, the sample became plastic, and the surface area increased. With the increase in microcracks, the samples exhibited plastic damage, and the process might have been in the rapid growth stage. The strain energy and consolidation fell sharply, and the elastic energy and kinetic energy were always low. As the fiber content increased, the scope of the fibers and the improved sand particles was expanded, the interaction forces were enhanced, and the energy release of the particles significantly increased after the particle consolidation was destroyed.

### 4.3. Polymer and Fiber Coupling Mechanism

**Figure 12:**
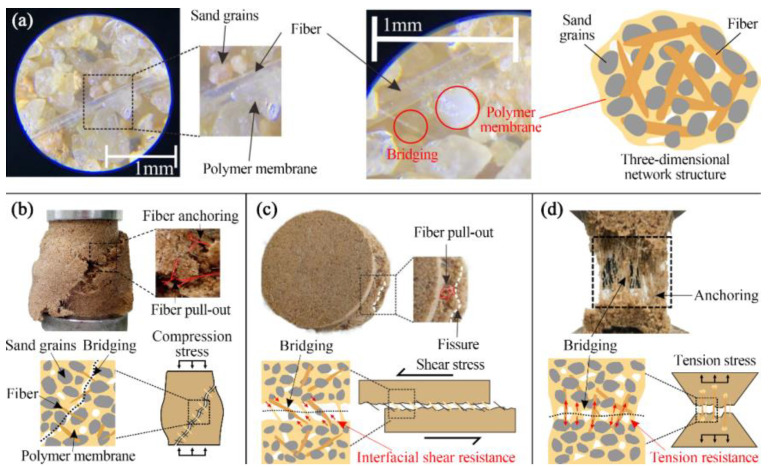
Mechanism diagram of polymer—fiber coupling: (**a**) Internal structure of the sample (**b**) failure of the compressive sample (**c**) failure of the direct shear sample (**d**) failure of the tensile sample.

The Mechanism diagram of the polymer—fiber coupling is shown in Figure 12. The major reason for the change in the macroscopic physical mechanical properties is the change in the microstructure of the macroscopic physical mechanics. There is no adhesion between the particles, the fibers are mixed into the sand, and a three-dimensional network is formed between the sand particles, which is the effect of the reinforcement. The organic polymer is adulterated with the sand soil, which fills the pore, parcel, and adhesive fiber, which effectively improves the entire sand soil. When organic polymer-fiber-modified sandy soil is subjected to an external load, the fiber network can transfer the scattered forces through the fascal-earth interface effect, expand the stress area, and reduce the concentration of stress. Moreover, the interlocking structure of the fiber network and sandy soil particles further limits the displacement and deformation of the sand particles, greatly improving the integrity of the sample, suppressing the deformation degree of the sample, and reducing the degree of specimen damage.

The resistance of grain and sand soil is determined to enhance the deformation capacity of modified sand soil, the fiber does not damage, the experiment is in the process of destruction, and the strength of the sample is determined mainly by the friction of the surface of the fiber and the friction of the sand particles, the adhesion of the fiber, the adhesion and friction between the joints and the different groups of the particles. The adhesion and package function of the organic polymer membrane and the aggregate group was damaged; the microcrack of the particles in the shear surface was rapidly expanded; the macroscopic crack was formed, and the change of the contact force chain structure of the improved sand soil was simulated; the reinforcement effect of some fibers was also broken by the large displacement, and the main failure forms were broken and separated. The three-dimensional network membrane is formed in the organic polymer membrane, and the polymer distribution of the fiber and sand particles of the organic polymer is enlarged, and the combination of the fiber and sand particles is formed, and the coupling effect of the fiber and sand particles is improved, and the occlusion degree of each group is enhanced, and a effective interlock structure is formed, so that the whole and mechanical properties of the samples are improved effectively.

## 5. Conclusions

In this study, the microdamage behavior of an organic polymer and fiber-treated sand in unconfined compressive strength, direct shear, and direct tensile tests was investigated using a series of numerical simulations. The following conclusions were drawn:

(1) The OPS and PF treatments improved the integrity of the sand, while a differential damage response was observed under varying test conditions. The bonding effect of the polymer resulted in multiple cracking paths and rough broken surfaces. The fibers served as anchors, stimulating lateral bulging and rugged failure of the specimens during loading.
(2) The maintenance of the mechanical strength in modified sand depends remarkably on the interparticle bonding state, which is affected by the force-chain network and crack distribution. The initial specimen cracking was concentrated and divided into the elastic, slow extension, rapid expansion, and residual stages. The increased homogeneous force transfer induced by the polymer membrane results in a greater range of particle motion and crack initiation. The fibers adhere to and confine the surrounding particles, resulting in arching force chains and dispersed/bending cracking.
(3) The external energy input to the modified sand is transformed into strain energy, which consists of elastic and bonding energies and breaking energy, which is the driving force for bonding deterioration. The energy evolution can be summarized in the compaction, elastic deformation, plastic failure, release, and residual stages. Polymer and fiber treatments increase energy capacity and improve release, thereby influencing deformation damage.
(4) Organic polymer and fiber mixed improvement of sand adopts a combination of chemical and physical improvement methods. The fiber was added to the sand to reinforce, whereas the organic polymer was added to the sand to wrap and fill. Organic polymers, fibers, and sand particles form an effective interlocking structure that enhances the integrity and mechanical properties of sand. This achievement provides a research basis for the application of numerical simulation technology in sand–soil composite improvements.

## Figures and Tables

**Table 1:** The physical parameters of used sand.

Type	S.g	*ρ*_d*max*_ (g/cm^3^)	*ρ*_d*min*_ (g/cm^3^)	*d*_10_ (mm)	*d*_30_ (mm)	*d*_60_ (mm)	*C*_u_	*C*_c_	Category (USCS)
value	2.66	1.7	1.32	0.12	0.22	0.36	3	1.12	SP

S.g is the specific gravity, *ρ*_d*max*_ is the maximum dry density, *ρ*_d*min*_ is the minimum dry density, *C*_u_ is the coefficient of uniformity, and *C*_c_ is the curvature of coefficient.

**Table 2:** Initial model parameter.

Parameters	Values	Data
Particles	Diameter of sand (mm)	0.07–2.0
	Density of sand (kg/m^3^)	1.50 × 10^3^
	Initial porosity of sand	0.10
	Diameter of fiber (mm)	0.04
	Density of fiber (kg/m^3^)	0.91 × 10^3^
	Coefficient of particle friction	0.5
	Damp	0.7
Parallel bond	Stiffness ratio	1.5
	Young’s and shear modulus (Pa)	0.65–2.58 × 10^5^
	Bond effective modulus (Pa)	0.4–2.0 × 10^5^
	Gap interval (mm)	5.0 × 10^−5^
	Cohesion (Pa)	0.35–2.25 × 10^5^
	Tensile strength (Pa)	0.70–4.50 × 10^5^
	Friction angle (°)	16–26
Liner bond	Stiffness ratio	1.5
	Young’s and shear modulus (Pa)	1.0 × 10^9^

## Data Availability

The data presented in this study are available on request from the corresponding author.
